# High-sensitivity ion detection at low voltages with current-driven organic electrochemical transistors

**DOI:** 10.1038/s41467-018-03932-3

**Published:** 2018-04-12

**Authors:** Matteo Ghittorelli, Leona Lingstedt, Paolo Romele, N. Irina Crăciun, Zsolt Miklós Kovács-Vajna, Paul W. M. Blom, Fabrizio Torricelli

**Affiliations:** 1 Department of Information Engineering, University of Brescia, via Branze 38, Brescia, 25123 Italy; 2 Max Planck Institute for Polymer Research, Ackermannweg 10, Mainz, 55128 Germany; 3 Present Address: Bruno Kessler Foundation, Centre for Materials and Microsystems, via Sommarive 18, 38123 Trento, Italy

## Abstract

Ions dissolved in aqueous media play a fundamental role in plants, animals, and humans. Therefore, the in situ quantification of the ion concentration in aqueous media is gathering relevant interest in several fields including biomedical diagnostics, environmental monitoring, healthcare products, water and food test and control, agriculture industry and security. The fundamental limitation of the state-of-art transistor-based approaches is the intrinsic trade-off between sensitivity, ion concentration range and operating voltage. Here we show a current-driven configuration based on organic electrochemical transistors that overcomes this fundamental limit. The measured ion sensitivity exceeds by one order of magnitude the Nernst limit at an operating voltage of few hundred millivolts. The ion sensitivity normalized to the supply voltage is larger than 1200 mV V^−1^ dec^−1^, which is the largest value ever reported for ion-sensitive transistors. The proposed approach is general and can be extended to any transistor technology, thus opening opportunities for high-performance bioelectronics.

## Abstract (web summary)

The organic electrochemical transistor is a type of transistor that modulates the channel current by the ion concentration and is thus explored for bio-applications. Here Ghittorelli et al. show a current-driven device configuration to increase the sensitivity by ten times than conventional approaches.

## Introduction

Ions dissolved in aqueous media play a fundamental role in plants, animals, and humans. Ions regulate the biological processes at the single cell scale, enable the propagation of electronic signals and maintain a suitable balance between the fluids of the extracellular and intracellular environments, which is extremely important for several processes including nerve impulses, hydration, muscle function, and the regulation of pH level^1–7^. The in situ quantification of the ion concentration in aqueous media is thus gaining significant interest in several emerging fields including biomedical diagnostics, environmental monitoring, healthcare products, water and food test and control, agriculture industry and security^8–17^.

Ion-sensitive field-effect transistors (ISFETs) are one of the most studied sensor-platforms for ion detection. In ISFETs the electrolyte solution is in contact with the insulator and the reference electrode acts as the gate^8,18–20^. The first ISFETs were fabricated using single-crystal silicon technology while, more recently, emerging technologies based on graphene, zinc oxide, silicon-nanowires, amorphous-oxides, and organics have been investigated^18,19,21–25^. Among them, organic technologies have triggered a great deal of interest because they are compatible with flexible large-area substrates, require simple processing, as for example spin coating or printing, allow simple functionalization through synthetic chemical modification, and the materials can be biocompatible, which is an essential prerequisite for in-vivo applications.

A fundamental challenge in applying organic transistors as ion sensors in aqueous solution is the stability, inherently due to the electrolysis. In order to avoid Faradaic leakage currents due to electrolysis, the maximum operating voltage has to be well below 1 V^17,26^. Low-voltage operation can be achieved by removing the insulating layer between the electrolyte and the organic semiconductor (OSC). Depending on the permeability of the OSC to the ions, two different classes of organic transistors can be obtained^17^. Ion-impermeable OSCs yield electrolyte-gated organic FETs (EGOFETs). In EGOFETs ions are compensated by charges (electrons or holes) accumulated into the OSC at the electrolyte-OSC interface, and an electric double-layer (EDL) is formed. The EDL capacitance is of the order of micro-Farads, ensuring a water-stable operation window^17,18,21,27^. On the other hand, ion-permeable OSCs yield organic electrochemical transistors (OECTs). In OECTs ions can drift into the OSC and compensate the induced charge carriers^28–30^. The electrochemical doping and de-doping processes modulate the conductivity of the OSC and are reversible^28–30^. Compared to EGOFETs, in OECTs the corresponding transconductance *g*_m_ = Δ*I*_D_/Δ*V*_G_ is extremely large because the interaction between ions and electronic charges takes place through the bulk of the OSC and not only at the electrolyte-OSC interface. Micro-scale OECTs show *g*_m_ > 10^−3^ S at very low gate bias, making them ideal ion-to-electron transducers^28,30–32^.

State of art OECTs show a current ion sensitivity of the order of tens μA dec^−1^ ^33,34^, that is one order of magnitude larger than that obtained with EGOFETs. The current variation is then converted into a voltage variation that, in turn, is related to the ion concentration by means of the Nernst equation^18,34,35^. When the current variation is converted into a voltage variation both OECTs and EGOFETs show comparable voltage ion sensitivity of the order of the Nernst limit, viz. 59 mV dec^−1^ at room temperature^34,35^. To improve the voltage ion sensitivity a possible approach is to increase the ratio between the electrolyte-OSC (*C*_d_) and gate-electrolyte (*C*_g_) capacitances (*C*_d_ /*C*_g_) by reducing *C*_g_^33^. Another approach relies on the use of a load resistor (*R*_L_) to directly convert the drain current into an output voltage resulting in an amplification *A* = *g*_m_*R*_L_^26^. Although in principle large *g*_m_ and *R*_L_ result in a large sensitivity, the output voltage should be compatible with the aqueous environment in the whole range of physiological ion concentration. The fundamental limitation of the aforementioned approaches is thus the stringent trade-off between the sensitivity, the operating range and the operating voltage.

Here we show low-voltage high-sensitivity ion detection with organic electrochemical transistors used in a current-driven inverter-like configuration. In the proposed approach the bias current sets the operating range of ion concentration and the sensitivity is not limited by the supply voltage, thus overcoming the fundamental limitation of state-of-art approaches. The measured voltage ion sensitivity exceeds by about one order of magnitude the Nernst limit in the case of supply voltage of 0.4 V, while it shows a five-fold increase in the case of supply voltage of 0.2 V in the ion concentration range 10^−4^–10^0^ M. The ion sensitivity normalized with respect to the supply voltage is larger than 1200 mV V^−1^ dec^−1^, which is more than one order of magnitude larger than the largest value ever reported for ion-sensitive transistors including ISFETs, EGOFETs, and OECTs. As relevant application example, a low-voltage ion-selective current-driven OECT with a voltage-normalized sensitivity as high as 1035 mV V^−1^ dec^−1^ is eventually demonstrated.

## Results

### Device structure and measurements

**Fig. 1:**
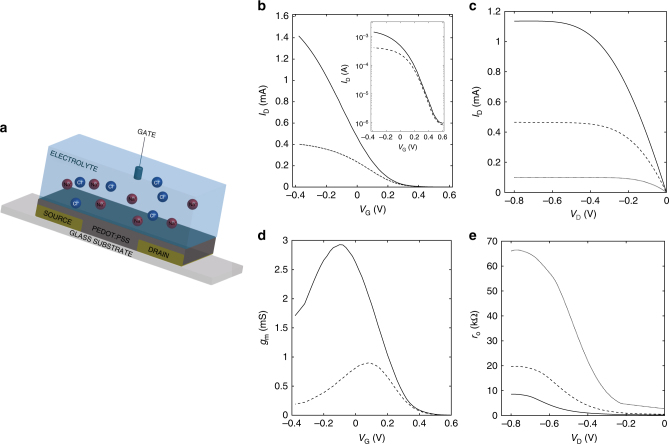
Transistor architecture and electrical characteristics. **a** Schematic structure of the fabricated OECTs. **b** Transfer characteristics at *V*_D_ = −0.1 V (dashed line) and *V*_D_ = −0.4 V (full line). The gate current is lower than 1 µA. **c** Output characteristics at *V*_G_ = 0.2 V (dotted line), *V*_G_ = 0 V (dashed line), and *V*_G_ = −0.2 V (full line). **d** Transconductance at *V*_D_ = −0.1 V (dashed line) and *V*_D_ = −0.4 V (full line). **e** Output resistance at *V*_G_ = 0.2 V (dotted line), *V*_G_ = 0 V (dashed line), and *V*_G_ = −0.2 V (full line). The gate is an Ag/AgCl pellet and the OECTs geometries are: *W* = 1000 µm, *L* = 50 µm, *t* = 50 nm. The NaCl concentration is 1 M

The device structure of the OECTs is shown in Fig. 1a. Gold source and drain contacts are evaporated through shadow mask on a glass substrate, and define the transistor length L and width W. As ion-permeable conducting polymer we deposited by spin coating Poly(3,4-ethylenedioxythiophene)-poly(styrenesulfonate) (PEDOT:PSS). The doping of PSS in PEDOT increases the conductivity of the polymer and enables the solubility of the PEDOT in water. Thus, PEDOT:PSS shows mixed electronic and ionic conductivity: PEDOT is the electronic semiconductor degenerately doped (*p*-type) by the ion-conducting electrical insulator PSS. The deposited PEDOT:PSS films have thicknesses (*t*) ranging from 25 to 100 nm. A 1.8 μm thick photoresist, deposited by spin coating and patterned photolithographically, insulates the gold electrodes from the electrolyte solution. As electrolyte, we used an aqueous solution of NaCl or KCl since sodium, potassium, and chloride are very relevant for biological processes. For example, they preserve suitable pressure and balance of various body fluids inside and between cells, as well as blood. Moreover, they are essential for maintaining suitable acidity into the body, passively balancing out the ions of tissue, blood, and organs^1,2,4,36,37^. A tungsten foil or Ag/AgCl pellet is immersed into the electrolyte and used as gate electrode. The former is a polarizable electrode, viz. it forms an EDL with the electrolyte, while the latter is a non-polarizable quasi-reference electrode. Further details on the transistors fabrication are provided in the Methods section. The transfer and output characteristics of a typical OECT are shown in Fig. 1b, c. The measurements show that the OECT operates in the water-stable operation window (|*V*_G_| < 1 V), the minimum current is about 10^−6^ A and the on/off current ratio is larger than 10^3^. The linear and saturation operating regions are clearly displayed in the output characteristics of Fig. 1c. Figure 1d shows the measured transconductance *g*_m_ = d*I*_D_/d*V*_G_ as a function of *V*_G_. The maximum *g*_m_ is about 3 mS at *V*_G_ = −0.1 V and *V*_D_ = −0.4 V, which agrees with state-of-art OECTs^28–30^. Figure 1e shows the measured output resistance *r*_o_ = (d*I*_D_/d*V*_D_)^−1^ as a function of *V*_D_. The maximum *r*_o_ is about 70 kΩ at *V*_D_ = −0.7 V and *V*_G_ = 0.2 V and it reduces to about 10 kΩ at *V*_G_ = −0.2 V.

### Current-driven OECT configuration

**Fig. 2:**
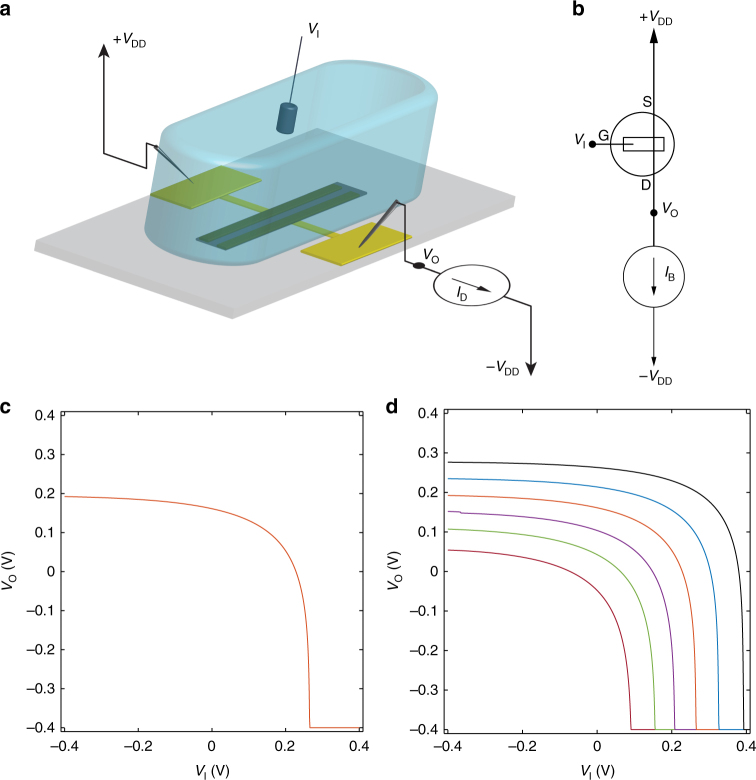
Current-driven OECT architecture and electrical characteristics. **a** Current-driven OECT configuration. **b** Schematic of the current-driven OECT. **c** Measured transfer characteristic of a current-driven OECT at *I*_B_ = 1.25 mA and *V*_DD_ = 0.4 V. **d** Measured transfer characteristics of a current-driven OECT by varying the bias current from *I*_B_ = 2.0 mA (brown curve) to *I*_B_ = 0.75 mA (black curve) with a step of −0.25 mA, *V*_DD_ = 0.4 V. The gate is a Ag/AgCl pellet and the OECTs geometries are: *W* = 1000 µm, *L* = 50 µm, *t* = 50 nm. The NaCl concentration is 1 M

The proposed current-driven OECT configuration is shown in Fig. 2a. The OECT is connected in series with a current generator resembling an inverter topology, as schematically depicted in Fig. 2b. The input voltage (*V*_I_) is applied to the gate (*V*_G_ = *V*_I_) while the output voltage (*V*_O_) is measured at the drain (*V*_D_ = *V*_O_). The bias current *I*_B_ is set by the current generator and the topology gives *I*_D_ = *I*_B_. A typical transfer characteristic (*V*_O_ – *V*_I_) with *I*_B_ = 1.25 mA is displayed in Fig. 2c. When the current-driven OECT is biased at negative *V*_I_ (e.g., *V*_I_ = −*V*_DD_), anions are injected into the PEDOT:PSS, the polymer is doped, and the OECT operates in linear region. The OECT output resistance *r*_o_ is small (Fig. 1e) and *V*_O_ = *V*_DD_–*I*_B_ *r*_o_ ≈ *V*_DD_. By increasing *V*_I_ anions are progressively extracted from the polymer and cations injected. Therefore, the polymer is de-doped, *r*_o_ increases, *V*_O_ lowers, and the OECT is eventually operated in saturation region. In the transfer characteristics *V*_O_–*V*_I_ the transition between the linear and the saturation region takes place when *V*_I_−*V*_O_ = *V*_P_, where *V*_P_ is the pinch-off voltage. As an example, in Fig. 2c the OECT saturation condition is achieved at *V*_I_ = + 0.22 V where *V*_O_ = −0.05 V and hence *V*_I_−*V*_O_ = + 0.27 V which, as readily visible in Fig. 1b, is equal to the pinch-off voltage *V*_P_ = + 0.27 V. In saturation, the transistor is pinched-off at the drain (output contact), *I*_D_ is almost independent of *V*_D_, *r*_o_ increases by more than one order of magnitude (Fig. 1e), and, as a consequence, V_O_ sharply drops to the minimum supply voltage –*V*_DD_. This is analogous to the zero-*V*_GS_ inverter topology^38^. Here the OECT is the driver and the current generator is the zero-*V*_GS_ load. When *V*_I_ is low the OECT operates in linear region and pulls *V*_O_ towards *V*_DD_. By increasing *V*_I_ the OECT becomes less conductive, *V*_O_ lowers and, in the case of Fig. 2c, at *V*_I_ ≥ 0.22 V the OECT is eventually operated in saturation, where *r*_o_ is large and *V*_O_ drops to –*V*_DD_. It is worth noting that the minimum supply voltage (–*V*_DD_) is set by means of the voltage-compliance of the current generator.

We define the switching voltage *V*_SW_ as the minimum *V*_I_ required to operate the OECT in saturation. The switching voltage provides meaningful information on the OECT parameters and can be analytically calculated as follows. The OECT current in the saturation regime reads^29,39^:

1 $$I_{\mathrm{D}} = {\beta}\left( {V_{\mathrm{S}} + V_{\mathrm{P}} - V_{\mathrm{G}}} \right)^2$$

where *β* = *W μ C*_v_ *t* (2 *L*)^−1^, *μ* is the charge carrier mobility, *C*_v_ is the volumetric capacitance, *V*_S_ is the source voltage, *V*_P_ = *q p*_0_ *C*_v_^−1^ is the pinch-off voltage, *q* is the elementary charge, and *p*_0_ is the initial charge carrier density in the conductive polymer before the application of the gate voltage. When the OECT is connected in the current-driven configuration *V*_S_ = *V*_DD_, *I*_D_ = *I*_B_, *V*_G_ = *V*_I_. Since the OECT starts to operate in saturation when *V*_I_ = *V*_SW_, the switching voltage can be analytically calculated by substituting *V*_G_ = *V*_SW_ in Eq. (1) and results:

2 $$V_{{\mathrm{SW}}} = V_{{\mathrm{DD}}} + V_{\mathrm{P}}-\left( {I_{\mathrm{B}}/{\beta}} \right)^{1/2}$$

Eq. (2) shows that *V*_SW_ depends on the main parameters of the current-driven configuration, i.e. the OECT physical and geometrical parameters (*V*_P_, *β*), the supply voltage *V*_DD_ and the bias current *I*_B_. Figure 2d shows the transfer characteristics *V*_O_–*V*_I_ of the current-driven OECT for several current biases *I*_B_. According with Eq. (2), by increasing *I*_B_ the switching voltage shifts to lower *V*_I_. This is because *V*_O_ gets lower at larger *I*_B_ and thus the OECT saturation occurs at a lower input voltage (i.e., *V*_SW_ reduces). Furthermore, Eq. (2) shows that *V*_SW_ also depends on the OECT parameters (viz. *V*_P_ and *β*) that, in turn, are related to the transistor geometry (namely *W*, *L*, *t*) and to the physical parameters of the conductive polymer and the electrolyte solution (namely *μ*, *C*_v_, and *p*_0_).

### Impact of the ion concentration

**Fig. 3:**
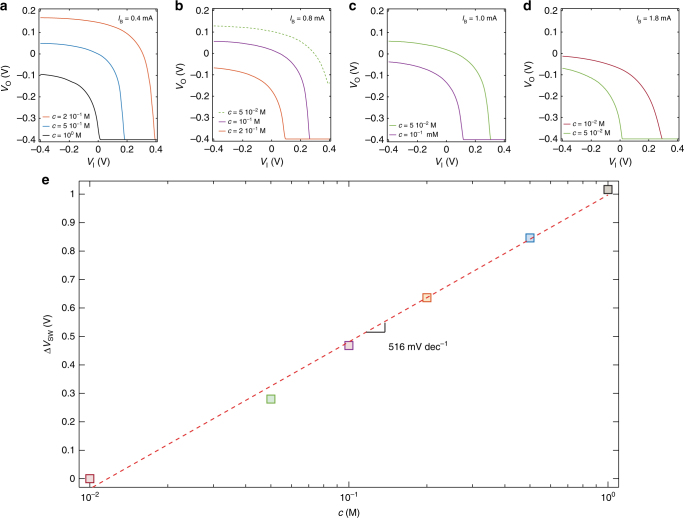
Current-driven OECT used as ion sensor. **a**–**d** Measured transfer characteristics of a current-driven OECT at several NaCl concentration *c*, *V*_DD_ = 0.4 V. **a** *I*_B_ = 0.4 mA, **b** *I*_B_ = 0.8 mA, **c** *I*_B_ = 1.0 mA, **d** *I*_B_ = 1.8 mA. **e** Cumulative switching voltage variation Δ*V*_SWi+1_ = Δ*V*_SWi_ + (*V*_SWi+1_−*V*_SWi_) as a function of ion concentration. The average sensitivity calculated by last-square linear approximation is of 516 mV dec^−1^. The gate is a tungsten foil and the OECTs geometries are: *W* = 1000 µm, *L* = 300 µm, *t* = 25 nm

In order to investigate the impact of the ion concentration on the electrical characteristics of the current-driven OECT, Fig. 3a–d show the measured *V*_O_−*V*_I_ as a function of the ion concentration. More in detail, Fig. 3a shows the *V*_O_–*V*_I_ characteristics of a current-driven OECT operated with a current bias *I*_B_ = 0.4 mA. When the ion concentration is *c* = 10^0^ M, the switching voltage is close to 0 V. By decreasing the ion concentration, *V*_SW_ shifts towards positive voltage. We found *V*_SW_ = 0.17 V and *V*_SW_ = 0.38 V with *c* = 5 10^−1^ M and *c* = 2 10^−1^ M, respectively. By further decreasing *c*, the switching voltage exceeds the supply voltage (*V*_DD_ = 0.4 V), and cannot be detected anymore since we sweep *V*_I_ in the range |*V*_I_| ≤ |*V*_DD_|. As shown in Eq. (2), by increasing the current bias *I*_B_ it is possible to shift *V*_SW_ back within the supply voltage range. This is confirmed in Fig. 3b which shows that the *V*_O_ – *V*_I_ characteristic obtained at *c* = 2 10^−1^ M is restored to *V*_SW_ ≈ 0.1 V. Consequently, the ion concentration can be decreased to *c* = 10^−1^ M resulting in a shift of the switching voltage to *V*_SW_ = 0.27 V. When *c* is further decreased to *c* = 5 10^−2^ M, *V*_SW_ cannot be detected anymore (Fig. 3b, dashed green line), and has to be restored within the supply voltage range by increasing *I*_B_. Similar considerations hold for the cases *I*_B_ = 1 mA (Fig. 3c) and *I*_B_ = 1.8 mA (Fig. 3d). It is worth to note that the current bias drives the OECT operation and sets *V*_SW_ within a given range of ion concentration, while the variation of the switching voltage is inherently related to the variation of the ion concentration. In other words, the ion concentration range is set by selecting *I*_B_ while *V*_SW_ is related to the value of *c*.

### Investigation of the ion sensitivity

In order to quantitatively assess the ion sensitivity of the proposed current-driven OECTs, we calculate the cumulative shift of the switching voltage, defined as Δ*V*_SWi+1_ = Δ*V*_SWi_ + (*V*_SWi+1_−*V*_SWi_), where *i* is the *i*-th ion concentration. Figure 3e shows Δ*V*_SW_ as a function of the ion concentration. The least-square linear approximation of the measured Δ*V*_SW_ (red dashed line) gives an average sensitivity of 516 mV dec^−1^ at a supply voltage of 0.4 V, that is about one order of magnitude larger than the theoretical Nernstian sensitivity of 59.16 mV dec^−1^. It is worth to note that the measured ion sensitivity of the current-driven OECT is almost constant in the whole range of ion concentration (Fig. 3e). For a given *I*_B_, a certain range of ion concentrations can be assessed, ensuring at the same time that the measured *V*_SW_ lies within the supply voltage. This condition can be achieved for any ion concentration and thus the sensitivity is not limited by the supply voltage. The possibility of selecting the ion concentration range by means of *I*_B_ enables to operate the current-driven OECT at extremely low supply voltages – fundamental to prevent Faradaic leakage currents due to water electrolysis—while maintaining at the same time a high ion concentration sensitivity (Δ*V*_SW_/Δ*c*). From Eq. (2), the ion concentration sensitivity can be calculated as follows:

3 $$\Delta V_{{\mathrm{SW}}}/\Delta c = + \Delta V_{\mathrm{P}}/\Delta c + \left[ {\left( {I_{\mathrm{B}}/\beta ^3} \right)^{1/2}/2} \right] \times\Delta \beta /\Delta c$$

Eq. (3) shows that the ion concentration sensitivity depends on the OECT parameters sensitivity, viz. Δ*V*_P_/Δ*c* and Δ*β*/Δ*c*.

**Fig. 4:**
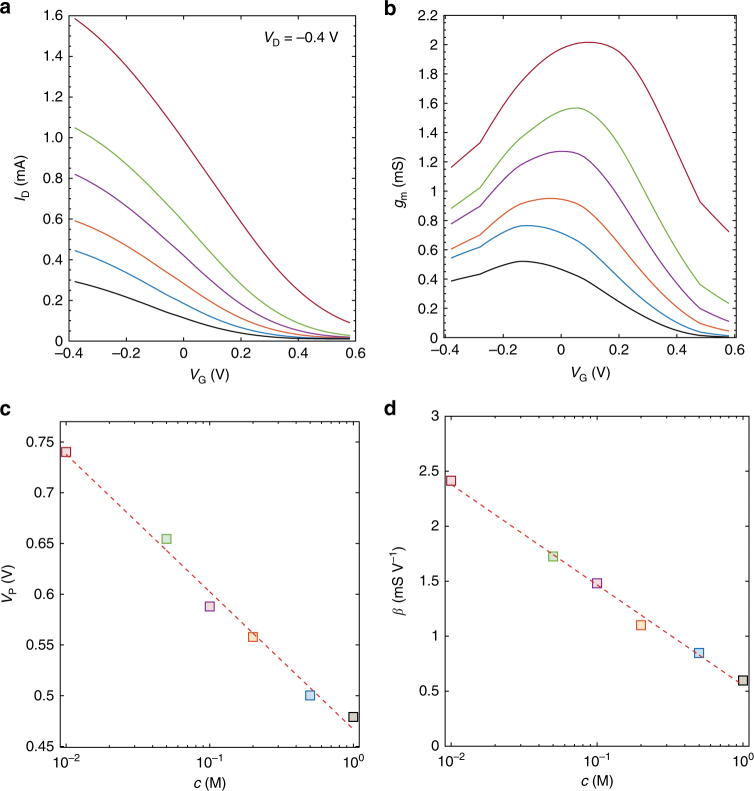
OECT characteristics and parameters as a function of the ion concentration. **a** Measured transfer characteristics of an OECT at several NaCl concentrations *c*, *V*_D_ = 0.4 V and **b** corresponding transconductance (*g*_m_). **c** Pinch-off voltage *V*_P_ as a function of the ion concentration. *V*_P_ is extracted by fitting the *I*_D_−*V*_G_ with the model^29,39^: *I*_D_ = 2*β* (−*V*_G_ + *V*_P_ + *V*_D_/2) V_D_. The crossing point (named *V*_X_) between the linear least square approximation of *I*_D_ and the *V*_G_-axis provides *V*_P_ = *V*_X_−*V*_D_/2. The average sensitivity is Δ*V*_P_/Δ*c* = −135 mV dec^−1^. **d** Current prefactor *β* as a function of the ion concentration. The average sensitivity is Δ*β*/Δ*c* = −0.9 mS V^−1^ dec^−1^. The gate is a tungsten foil and the OECTs geometries are: *W* = 1000 µm, *L* = 300 µm, *t* = 25 nm

To gain more insight on the origin of the high ion sensitivity of the current-driven OECT configuration, it is crucial to estimate the OECT parameters sensitivity. Figure 4a, b show the measured transfer characteristics and transconductances as a function of the ion concentration, ranging from 10^−2^ to 10^0^ M, at *V*_D_ = −0.4 V. The measured transfer characteristics shift toward negative voltages and the transconductances get lower by increasing the ion concentration. By reproducing the measurements with the model proposed by Bernards and Malliaras (Eq. 1)^29,39^, we estimated the OECT parameters *V*_P_ and *β* at several ion concentrations. Figure 4c–d show that both *V*_P_ and *β* linearly depend on the logarithm of the ion concentration and we found Δ*V*_P_/Δ*c* = −135 mV dec^−1^ and Δ*β*/Δ*c* = − 0.9 mS V^−1^ dec^−1^. It is worth noting that Δ*V*_P_/Δ*c* agrees with state-of-art OECTs operated with polarizable gate electrode (e.g., tungsten).

### Low-voltage operation

In order to evaluate the effectiveness of the proposed approach at lower voltage, we investigated current-driven OECTs operated at a supply voltage as low as *V*_DD_ = 0.2 V. We note that this operating voltage is significantly lower than that typically used for OECTs^28,30,33^, OECT-based ion sensors^34^ and voltage amplifiers^26^. To optimize the operation at low voltages, we replaced the tungsten gate with a Ag/AgCl gate electrode. This minimizes the voltage drop at the gate electrode/electrolyte interface at the expense of a slightly reduction of Δ*V*_P_/Δ*c*. Moreover, the OECT geometries have been optimized to warrant the detection of one order of magnitude of concentration for a given current bias *I*_B_.

**Fig. 5:**
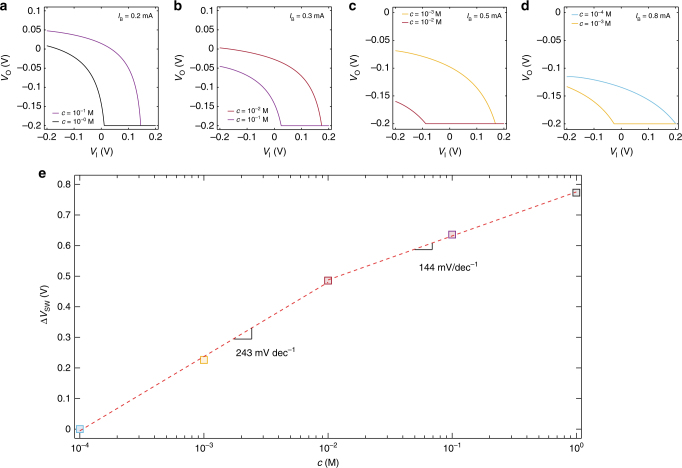
Ultra-low voltage current-driven OECT. **a**–**d** Measured transfer characteristics of the current-driven OECT at several NaCl concentration *c*, *V*_DD_ = 0.2 V. **a** *I*_B_ = 0.2 mA, **b** *I*_B_ = 0.3 mA, **c** *I*_B_ = 0.5 mA, **d** *I*_B_ = 0.8 mA. **e** Cumulative switching voltage variation Δ*V*_SWi+1_ = Δ*V*_SWi_ + (*V*_SWi+1_−*V*_SWi_) as a function of the ion concentration. The average sensitivity is of 144 mV dec^−1^ in the range 10^−2^–10^0^ M and 243 mV dec^−1^ in the range 10^−4^–10^−2^ M. The gate is a Ag/AgCl pellet and the OECTs geometries are: *W* = 1000 µm, *L* = 100 µm, *t* = 50 nm

Figure 5a–d show the measured *V*_O_−*V*_I_ of the ultra-low voltage current-driven OECT as a function of the ion concentration. More in detail, Fig. 5a shows the *V*_O_–*V*_I_ characteristics of a current-driven OECT operated with a current bias *I*_B_ = 0.2 mA. When the ion concentration is *c* = 10^0^ M, the switching voltage is close to 0 V. By decreasing the ion concentration, V_SW_ shifts towards positive voltages. We found *V*_SW_ = 0.15 V with *c* = 10^−1^ M. Further decreasing *c*, the switching voltage exceeds the supply voltage (*V*_DD_ = 0.2 V), and cannot be detected anymore. By increasing the current bias *I*_B_ it is possible to shift *V*_SW_ back within the supply voltage range. Figure 5b shows the *V*_O_–*V*_I_ characteristics of a current-driven OECT operated with a current bias *I*_B_ = 0.3 mA. When the ion concentration is *c* = 10^−1^ M, the switching voltage is restored to *V*_SW_ ≈ 0 V. Consequently, the ion concentration can be decreased to *c* = 10^−2^ M resulting in a shift of the switching voltage to *V*_SW_ = 0.17 V. Similar considerations hold for the cases *I*_B_ = 0.5 mA (Fig. 5c) and *I*_B_ = 0.8 mA (Fig. 5d).

To quantitatively assess the ion sensitivity of the ultra-low voltage current-driven OECTs, Fig. 5e shows Δ*V*_SW_ as a function of the ion concentration in the range 10^−4^–10^0^ M. The linear least-square approximation of the measured Δ*V*_SW_ (red dashed line) gives an average sensitivity of 243 mV dec^−1^ in the ion concentration range 10^−4^–10^−2^ M and of 144 mV dec^−1^ in the range 10^−2^ to 10^0^ M, at a supply voltage as low as 0.2 V.

### Ion-selective operation

As a relevant application example, high-sensitivity ion-selective detection is demonstrated by endowing the current-driven OECT with an ion-selective membrane. According with previous works^34,35^ an ion-selective membrane is fabricated and used to separate an inner electrolyte in direct contact with the OECT from the analyte. The inner and the analyte electrolytes are confined in two wells, one stacked on the top of the other, and separated by the ion-selective membrane. Further details are provided in the Methods section. To directly compare the current-driven approach with state-of-art ion-selective OECTs, an Ag/AgCl pellet is used as gate electrode, and a membrane selective to K^+^ ions is fabricated as described in^34^. A 10^−2^ M KCl electrolyte is used as inner filling solution while KCl or NaCl at several concentrations are used as analyte solution.

**Fig. 6:**
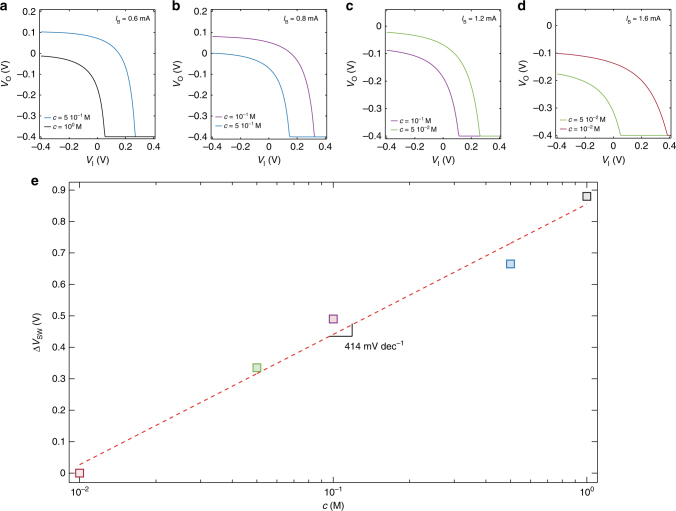
Ion-selective current-driven OECT. **a**–**d** Measured transfer characteristics of an ion-selective current-driven OECT at several KCl concentrations *c*, *V*_DD_ = 0.4 V. **a** *I*_B_ = 0.6 mA, **b** *I*_B_ = 0.8 mA, **c** *I*_B_ = 1.2 mA, **d** *I*_B_ = 1.6 mA. **e** Cumulative switching voltage variation Δ*V*_SWi+1_ = Δ*V*_SWi_ + (*V*_SWi+1_−*V*_SWi_) as a function of the ion concentration. The average sensitivity is 414 mV dec^−1^. The gate is a Ag/AgCl pellet, the membrane is selective to K^+^ ions, and the OECTs geometries are: *W* = 1000 µm, *L* = 300 µm, *t* = 100 nm

Figure 6a–d show the measured *V*_O_−*V*_I_ of an ion-selective current-driven OECT as a function of the ion concentration. According with the previous analysis, *V*_SW_ shifts to more positive voltages when the ion concentration is reduced and it is possible to shift *V*_SW_ back within the supply voltage range by increasing the current bias *I*_B_. This ensures, at the same time, both high-sensitivity and low-voltage operation. To evaluate the ion sensitivity of ion-selective current-driven OECTs, Fig. 6e shows Δ*V*_SW_ as a function of the ion concentration. The linear least-square approximation of the measured Δ*V*_SW_ (red dashed line) gives an average sensitivity of 414 mV dec^−1^ at a supply voltage equal to 0.4 V, which is more than 8-fold larger than the ion sensitivity obtained by using an OECT^34^. Moreover, it is worth to note that the sensitivity obtained with the ion-selective membrane is close to the value obtained without the ion-selective membrane.

Finally, the selectivity is investigated by using NaCl as analyte solution. Supplementary Figs. 1a, b show the measured *V*_O_−*V*_I_ as a function of the ion concentration and at several *I*_B_. Independently of the NaCl concentration *V*_O_ is almost constant in the supply voltage range and no switching occurs. This confirms that the membrane is selective to K^+^ ions, and the current-driven OECT configuration can be successfully used for high sensitivity and selective ion detection.

## Discussion

The current-driven OECT configuration provides, at the same time, low-voltage operation and high sensitivity. The sensitivity, Δ*V*_SW_/Δ*c*, depends on the sum of two contributions (Eq. 3), i.e., Δ*V*_SW_/Δ*c* = Δ*V*_P_/Δ*c* + Δ*V*_β_ /Δ*c*, where Δ*V*_β_ /Δ*c* = *k* x Δ*β*/Δ*c* and *k* = (*I*_B_/*β*^3^)^1/2^/2. Since in the current-driven OECT configuration both *V*_P_ and *β* decrease by increasing the ion concentration, the terms Δ*V*_β_/Δ*c* and Δ*V*_P_/Δ*c* sum up resulting in an improved voltage ion sensitivity with respect to that obtained with conventional OECT operation, where the voltage sensitivity is limited to only Δ*V*_P_/Δ*c*^33^.

The current-driven OECTs overcome these fundamental limitations by adding a very relevant contribution to the voltage ion sensitivity, i.e. Δ*V*_β_ /Δ*c* = *k* × Δ*β*/Δ*c*, that can be enhanced by means of the OECT design parameters, viz. channel width and length, semiconductor thickness, mobility, and volumetric capacitance. For example, the results presented in Figs. 3 and 4 show that current-driven OECTs operated at *I*_B_ = 0.8 mA and at *c* = 2 10^−1^ M gives *k* ≈ 400 and therefore Δ*V*_β_ /Δ*c* ≈ 360 mV dec^−1^, which yield Δ*V*_β_ /Δ*c* ≈ 3 × Δ*V*_P_/Δ*c*.

Interestingly, ultra-low voltage current-driven OECTs show a voltage ion sensitivity Δ*V*_SW_/Δ*c* = Δ*V*_P_/Δ*c* + Δ*V*_β_ /Δ*c* larger at smaller concentrations (Fig. 5e). This can be explained as follows. Supplementary Fig. 2c shows that *V*_P_ linearly depends on the logarithm of the ion concentration while Supplementary Fig. 2d shows that *β* has a double linear dependence on the logarithm of the ion concentration. We found that Δ*V*_P_/Δ*c* = −98 mV dec^−1^ in the whole concentration range, while Δ*β*/Δ*c* = −0.28 mS V^−1^ dec^−1^ in the concentration range 10^−4^–10^−2^ M and Δ*β*/Δ*c* = −0.10 mS V^−1^ dec^−1^ in the range 10^−2^–10^0^ M. Consequently, Δ*V*_β_/Δ*c* = *k* × Δ*β*/Δ*c* is larger in the concentration range 10^−4^–10^−2^ M, and this explains the larger ion sensitivity at smaller concentrations. The variation of *β* as a function of the ion concentration *c* could be related to a variation of *C*_V_ and/or *μ*. To investigate this point, we extracted *C*_V_ and *μ* as a function of the ion concentration. Taking advantage of electrochemical impedance spectroscopy measurements (Supplementary Fig. 3) we found that *C*_V_ is independent of *c* and results *C*_V_ = 39.38 ± 1.76 F cm^−3^, in good agreement with state-of-art PEDOT-PSS OECTs^28–30^. Then, we extracted *μ* as a function of *c* from the measured *I*_D_-*V*_G_ (Supplementary Fig. 4). We found that *μ* decreases by increasing *c*, thus explaining the *β*–*c* characteristic displayed in the Supplementary Fig. 2d.

The proposed approach is compared with several ion-sensitive transistor technologies in Table 1. The voltage ion sensitivity of the current-driven OECT configuration outperforms silicon, metal-oxide and electrolyte-gated organic field-effect transistors. It is only surpassed by ZnO double gate transistors^18,21,40^, which require a more complex multi-layer fabrication and a quite large supply voltage (up to 20 V) inherently related to the small gate capacitance. In order to fairly compare the different transistor technologies, the voltage ion sensitivity is normalized with respect to the supply voltage. As shown in Table 1, the normalized voltage ion sensitivity *A*v_I_ = (Δ*V*_SW_/Δ*c*)/*V*_DD_ obtained with current-driven OECTs at *V*_DD_ = 0.2 V and *V*_DD_ = 0.4 V results *A*v_I_ = 1215 mV V^−1^ dec^−1^ and *A*v_I_ = 1290 mV V^−1^ dec^−1^, respectively. These values are more than one order of magnitude larger than those reported for state-of-art ion-sensitive transistors including Si ISFETs, ZnO single and double gate FETs, a-IGZO double gate FETs, Si nanowire FETs, graphene FETs, EGOFETs, and OECTs.

**Table 1:** Sensitivity comparison

Technology	Ion	Δ*I*/Δ*c* (µA dec^−1^)	Δ*V*/Δ*c* (mV dec^−1^)	*V*_DD_ (V)	*A*_VI_ (mV V^−1^ dec^−1^)	Reference
Si ISFET	pH	60	53	2.5	21.2	^23^
ZnO—single gate	pH	90	55	2	27.5	^23^
ZnO—double gate	pH	0.012	2250	20	112.5	^18,21,40^
a-IGZO—double gate	pH	—	160	5	32.0	^25^
Si NanoWire	pH	1	—	40	—	^24^
Si NanoWire—double gate	pH	—	220	7	31.43	^19^
Graphene FET	pH	—	49	0.4	122.5	^22^
Ion-selective P3HT EGOFET	Na^+^	0.5	62	0.5	124.0	^35^
Ion-selective OECT	K^+^	47	48	0.4	120.0	^34^
OECT	Na^+^ K^+^ H^+^	20	135	1.25	108.0	^33^
OECT	Na^+^	780	135	0.4	337.5	This work
OECT	Na^+^	270	98	0.4	245.0	This work
Current-driven OECT	Na^+^	—	516	0.4	1290.0	This Work
Current-driven OECT	Na^+^	—	243	0.2	1215.0	This Work
Ion-selective current-driven OECT	K^+^	—	414	0.4	1035.0	This work

The table compares several technologies by considering the type of ion, the current sensitivity (ΔI/Δ*c*), the voltage sensitivity (Δ*V*/Δ*c*), the supply voltage (*V*_DD_), and the supply voltage-normalized voltage sensitivity *A*_VI_ = (Δ*V*/Δ*c*)/*V*_DD_

The current-driven OECT approach ensures, at the same time, low-voltage operation and high sensitivity in a wide range of ion concentrations. In contrast to state-of-art ion sensors where the input ion concentration is converted in an output voltage^26^, the current-driven OECT configuration is based on the reading of the switching voltage, which is positioned inside the supply voltage range by properly selecting the bias current. Therefore, the current-driven OECT approach exploits the large transconductance of OECTs in a completely different fashion with respect to standard voltage amplifier configurations^26^ and breaks the intrinsic trade-off between sensitivity and operating voltage.

In summary, the current-driven OECT approach shows that is possible to dramatically enhance the ion sensitivity of OECTs while maintaining a low-voltage operation. The key ingredients are the large transistor transconductance that reflects in the large amplification and the bias current that enables to select the operating range and ensures the low-voltage operation.

The measured voltage ion sensitivity exceeds by about one order of magnitude the Nernst limit in the case of supply voltage of 0.4 V, while it shows a five-fold increase in the case of supply voltage of 0.2 V. The ion sensitivity normalized with respect to the supply voltage is larger than 1200 mV V^−1^ dec^−1^, which is the largest value ever reported for ion-sensitive transistors.

The proposed approach is general and could be extended to other electrochemical and field-effect transistor technologies. In addition, current-driven OECTs allow simple and low-cost fabrication on flexible substrates, opening new opportunities for high-performance conformable and widespread organic bioelectronics.

## Methods

### Device fabrication

Glass slides were cleaned by sonication in acetone, isopropyl alcohol, and deionized water, followed by drying and UV ozone cleaning. 100 nm thick gold source/drain contacts were evaporated through a shadow mask defining the device channel length. An adhesion layer of 5 nm of chromium has been previously deposited. For the preparation of the organic semiconductor films, 20 ml of aqueous dispersion of poly(3,4-ethylenedioxythiophene): poly(styrene sulfonic acid) PEDOT:PSS (PH-500 from Heraeus Clevios GmbH) and 1 ml of Dimethyl sulfoxide (DMSO) were mixed and sonicated before spin coating^41^. The films were subsequently baked at 140 °C for 1 h and immersed in deionized water to remove any excess low-molecular-weight compounds. A 1.8 μm AZ1518 positive photoresist purchased form Microchemicals GmbH and used as received, was spin coated and photolithographically patterned on top of electrodes in order to avoid unwanted electrochemical reaction of the gold electrodes.

### Ion-selective membrane

The PVC-based membrane was prepared by mixing high molecular weight PVC (36.5 wt.%) with potassium ionophore III (2.5 wt.%), potassium tetrakis(4-chlorophenyl-)borate (0.5 wt.%) and diisodecyl adipate (60.5 wt.%) in THF (500 mg/5 mL). The mixture was drop-casted onto a glass slide and dried at room temperature. A rubber ring defined the dimension of the membrane and provided the necessary stability for further transfer^34^. ISM-OECT: A small PDMS-well, containing the inner solution (10^−2^ M KCl), was used as a mechanical support for the K^+^-selective membrane. On top of the ion-selective membrane, it is placed a small chamber (transwell filter, pore size 0.4 μm) with the analyte solution at various concentrations. A quasi-reference Ag/AgCl electrode was immersed into the analyte solution and used as gate electrode (1 mm pellet, Warner Instruments).

### Electrical characterization

All characterization was performed using a solution of NaCl or KCl in DI water as the electrolyte at several different concentrations and a tungsten foil or an Ag/AgCl pellet (Warner Instruments) as the gate electrode. The DC electrical characteristics of the OECTs were measured with a Keithley 4200 SCS parameter analyser. Electrochemical Impedance Spectroscopy as a function of the ion concentration are performed in the three-electrode configuration, where two Ag/AgCl pellets serve as reference and counter electrodes. The source and drain electrodes are shorted together, and the OECT channel is used as the working electrode. We used the National Instruments PXI-1042 system, equipped with a PXI-5421 arbitrary wave-function generator, a PXI-5112 oscilloscope and custom Labview software for the control of the PXI system.

### Data availability

The data that support the findings of this study are available from the corresponding author on reasonable request.

## Electronic supplementary material

Supplementary Information
