# Supplementary material for: High-sensitivity ion detection at low voltages with current-driven organic electrochemical transistors
Source: Nat Commun. 2018 Apr 12;9:1441. doi: 10.1038/s41467-018-03932-3 (PMC5897342; doi:10.1038/s41467-018-03932-3)
Supplement: Supplementary file 1 — Supplementary Information [file 41467_2018_3932_MOESM1_ESM.pdf]

Supplementary Information

High-sensitivity ion detection at low voltages with current-driven organic electrochemical transistors

Matteo Ghittorelli, Leona Lingstedt, Paolo Romele, N. Irina Crăciun, Zsolt Miklós Kovács-Vajna, Paul W. M. Blom & Fabrizio Torricelli

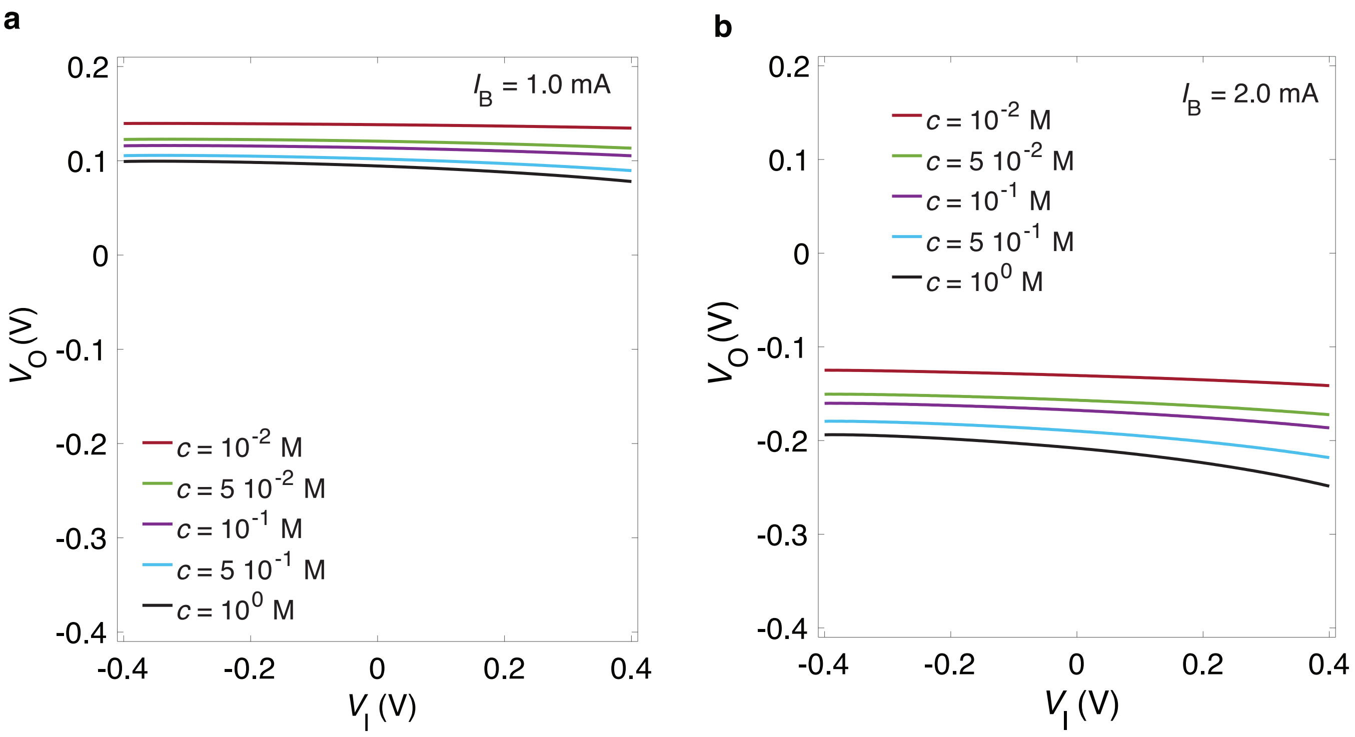

Supplementary Figure 1 | Measured transfer characteristics (V_O-V_I) of the current-driven OECT at several NaCl concentration *c*, V_{DD} = 0.4 V. (**a**) I_B = 1 mA, and (**b**) I_B = 2.0 mA. The membrane, placed in between the inner and the analyte solutions, is selective to K^+ ions. Independently of the NaCl concentration V_O is almost constant and no switching is displayed. The gate is a Ag/AgCl pellet and the OECTs geometries are: W = 1000 µm, L = 300 µm, t = 100 nm.

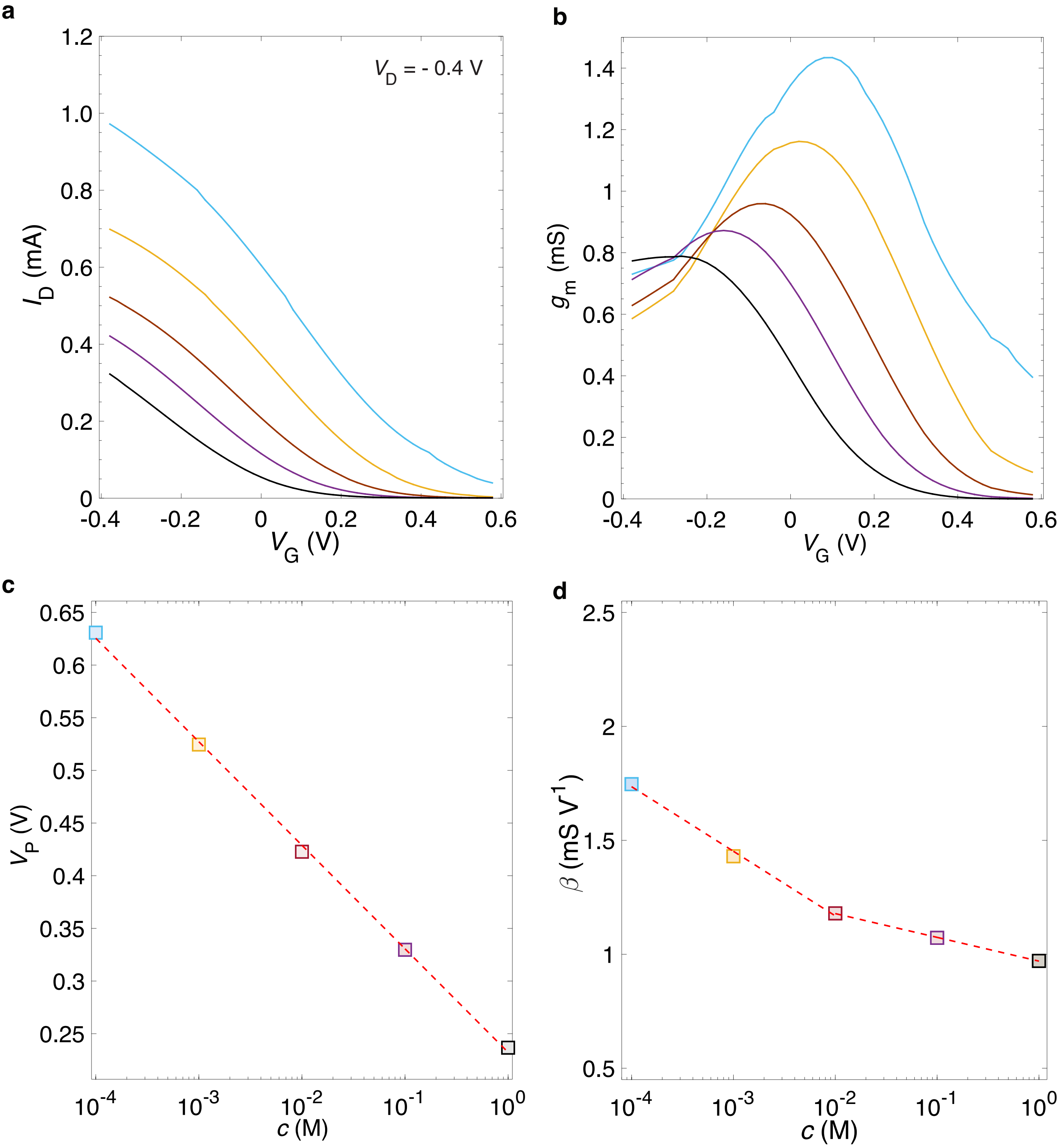

Supplementary Figure 2 | Characteristics and parameters of OECTs operated at ultra-low voltage. (**a**) Measured transfer characteristics of the OECT at several ion concentrations, V_D = 0.4 V and (**b**) corresponding transconductance (g_m). (**c**) Pinch-off voltage V_P as a function of the ion concentration. The average sensitivity is $\Delta V_P/\Delta c$ = - 98 mV dec^{-1}. (**d**) Current prefactor β as a function of the ion concentration. The average sensitivity is $\Delta\beta/\Delta c$ = - 0.28 mS V^{-1} dec^{-1} in the concentration range 10^{-4} to 10^{-2} M and $\Delta\beta/\Delta$c = - 0.10 mS V^{-1} dec^{-1} in the range 10^{-2} to 10^{0} M. The gate is a Ag/AgCl pellet and the OECTs geometries are: W = 1000 µm, L = 100 µm, t = 50 nm.

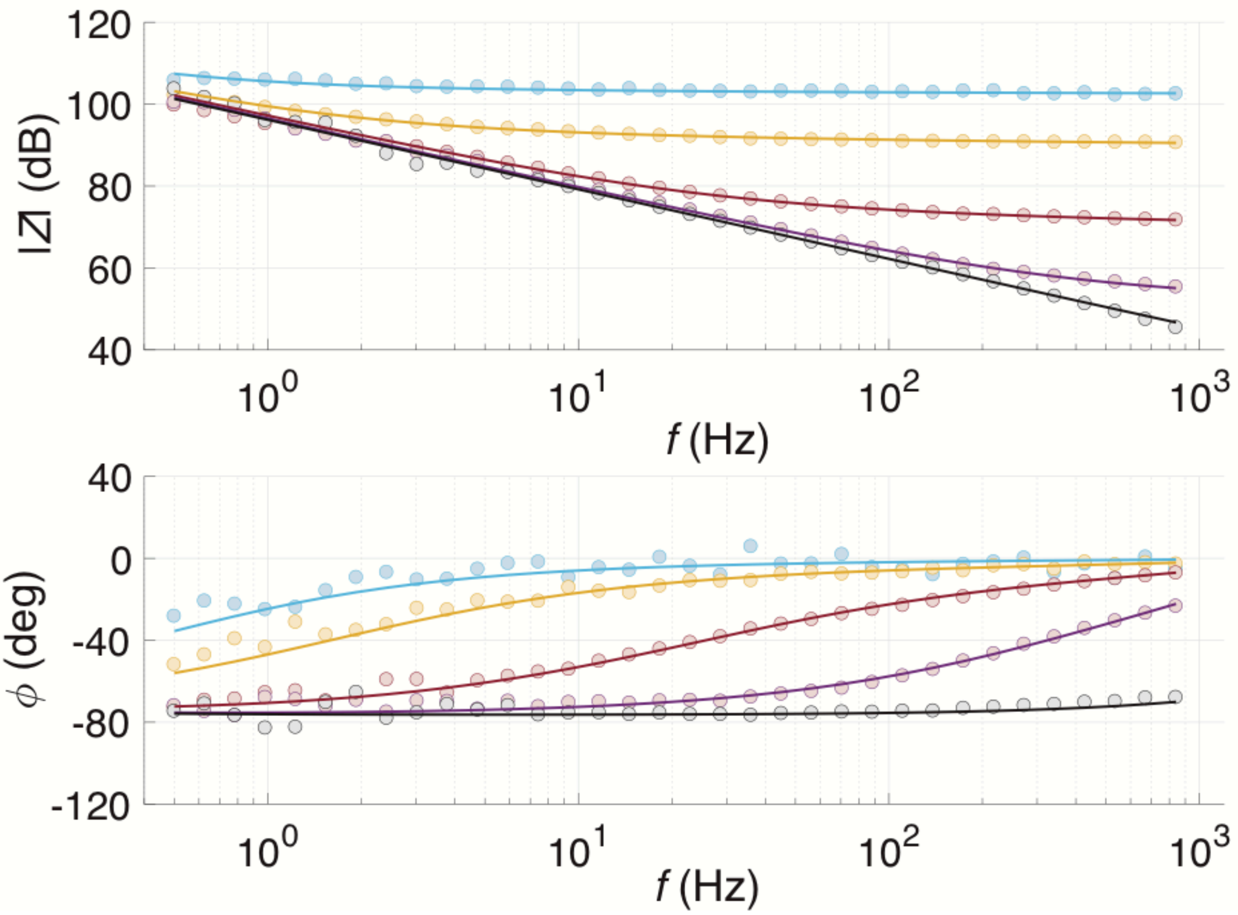

Supplementary Figure 3 | OECT impedance spectra as a function of ion concentration c = [10^{-4} 10^{-3} 10^{-2} 10^{-1} 10^{0}] M. In order to extract the volumetric capacitance C_V, for each ion concentration we modelled the measured impedance as a function of the frequency with the Randles equivalent circuit. According with Ref. 1, in the case of an OECT the Randles circuit is composed by a series resistor R_s, and a parallel of a resistor R_p and a capacitor C. In this model, R_s is the electrolyte resistance, R_p depends on the Faradaic reactions at the working electrode, and C is the capacitance. The OECT volumetric capacitance is then calculated as $C_v = C\, v^{-1}$ where $v = W\, L\, t$ is the total volume of PEDOT:PSS. In the case of analysis W = 1000 µm, L = 800 µm, and t = 100 nm. We reproduced the measurements (symbols) with the model (full line) and we found that for all ion concentrations R_p > 5 MΩ, R_s is proportional to the inverse of the ion concentration, in agreement with the Debye-Hückel-Onsager theory [3], and the volumetric capacitance is independent of the ion concentration and results C_V = 39.38 ± 1.76 F cm^{-3}.

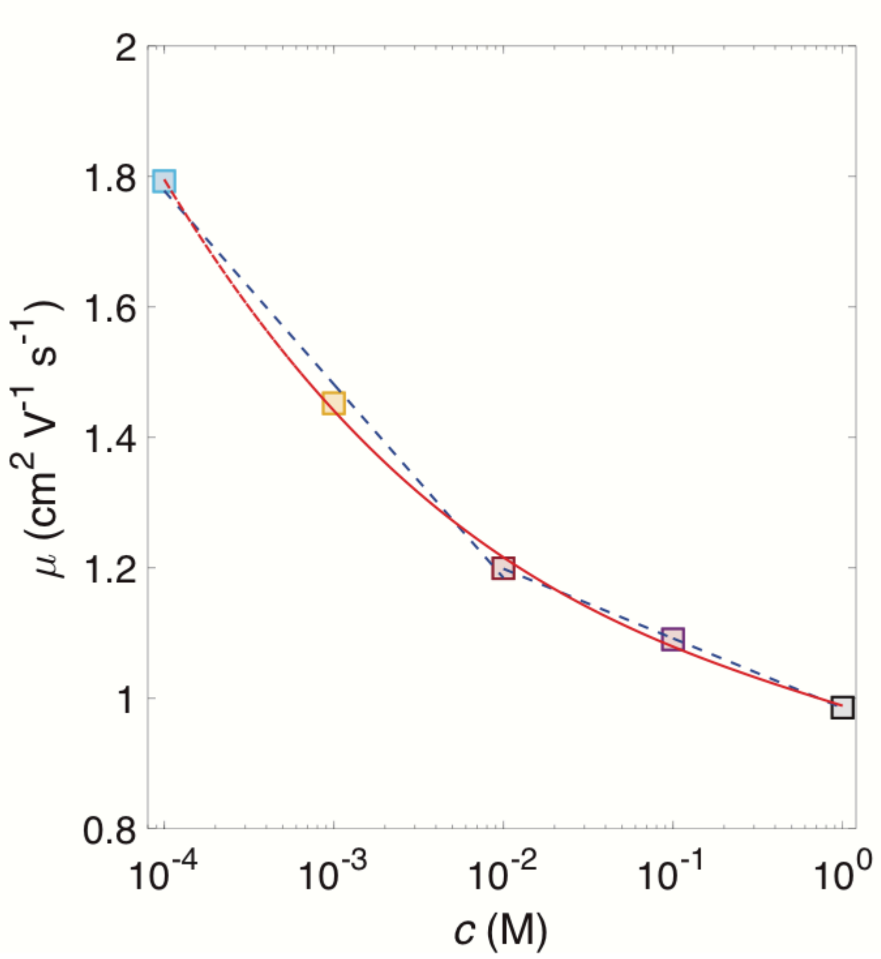

Supplementary Figure 4 | Mobility as a function of the ion concentration. Symbols are calculated from the measurements, dashed blue line is a simple double logarithmic approximation and red line is a third-order approximation. Lines are used only as guides for eyes. According with the model proposed by Bernards and Malliaras[1,3], when the transistor is operated in the linear regime the mobility can be extracted from the transfer characteristics as follows $\mu(c) = g_{m*}\ L\ (W\ C_V\ t\ V_D)^{-1}$ where $g_{m*} = \max(\Delta I_D/\Delta V_G)$ is the maximum transconductance. The mobility decreases by increasing the ion concentration. This could be interpreted as follows. In PEDOT:PSS the charge transport takes place in PEDOT nanocrystals surrounded by an amorphous PEDOT:PSS matrix[4]. Ions easily penetrate the amorphous phase of PEDOT:PSS and interacts with the PEDOT nanocrystals[5,6]. This situation is analogous to the case where ions enter a semicrystalline organic semiconductor. In this case, it has been found that the overall semiconductor mobility gets lower by increasing the ion concentration because the highly oriented fraction of the semiconducting polymer reduces[7].

Supplementary References

1. Rivnay, J. *et al.* High-performance transistors for bioelectronics through tuning of channel thickness. *Sci. Adv.* 1, e1400251 (2015).
2. Cetin, M. Electrolytic conductivity, Debye-Hückel theory, and the Onsager limiting law. *Phys. Rev. E* 55, 2814–2817 (1997).
3. Bernards, D. A. & Malliaras, G. G. Steady-state and transient behavior of organic electrochemical transistors. *Adv. Funct. Mater.* 17, 3538–3544 (2007).
4. Honma, Y. *et al.* Mesoscopic 2D Charge Transport in Commonplace PEDOT:PSS Films. *Adv. Electron. Mater.* 1700490, 1–6 (2018).
5. Inal, S., Malliaras, G. G. & Rivnay, J. Optical study of electrochromic moving fronts for the investigation of ion transport in conducting polymers. *J. Mater. Chem. C* 4, 3942–3947 (2016).
6. Rivnay, J. *et al.* Structural control of mixed ionic and electronic transport in conducting polymers. *Nat. Commun.* 7, 1–9 (2016).
7. Guardado, J. O. & Salleo, A. Structural Effects of Gating Poly(3-hexylthiophene) through an Ionic Liquid. *Adv. Funct. Mater.* 27, 1701791 (2017).
